# Cannabidiol for the treatment of positive and negative symptoms in schizophrenia spectrum disorders: a systematic review and meta-analysis

**DOI:** 10.1007/s00406-026-02249-3

**Published:** 2026-04-28

**Authors:** Antonio Di Francesco, Pierfelice Cutrufelli, Cecilia Chiarenza, Luca Zambuto, Carmen Concerto, Ludovico Mineo, Antonino Petralia, Marco Catalfo, Alessandro Rodolico, Myrto Samara, Stefan Leucht, Filippo Caraci, Maria Salvina Signorelli

**Affiliations:** 1 Department of Clinical and Experimental Medicine, Institute of Psychiatry, University of Catania, Catania, Italy; 2 Department of Psychiatry and Psychotherapy, Klinikum rechts der Isar, School of Medicine, Technical University of Munich, Munich, Germany; 3 Department of Psychiatry, Faculty of Medicine, University of Thessaly, 41334 Larissa, Greece; 4 Department of Drug and Health Sciences, University of Catania, Catania, Italy; 5 Oasi Research Institute-IRCCS, Unit of Translational Neuropharmacology and Translational Neurosciences, Troina, Italy

**Keywords:** Schizophrenia, Cannabis, CBD, THC, Psychosis, Meta-analysis

## Abstract

**Background:**

Cannabidiol (CBD) has shown therapeutic potential as an antipsychotic in preclinical studies, although the pharmacodynamics profile remains to be fully explained. Current research focuses on CBD as an adjunctive strategy, a design that allows addressing residual symptoms while avoiding the ethical and clinical risks associated with discontinuing standard antipsychotic medication. This systematic review and meta-analysis evaluated the efficacy of CBD as an adjunctive therapy for positive and negative symptoms in schizophrenia spectrum disorders.

**Methods:**

PubMed and CENTRAL were searched up to May 14, 2025. We included randomized controlled trials (RCTs) comparing CBD augmentation to placebo in adults with these disorders. Primary outcomes were changes on the Positive and Negative Syndrome Scale (PANSS).

**Results:**

Five RCTs were included. Meta-analysis showed a statistically significant advantage for CBD over placebo for PANSS total scores (Mean Difference, MD -1.91; 95% Confidence Interval, CI -3.726 to -0.102), positive subscale (MD -1.304; CI -1.917 to -0.692), and general subscale (MD -1.094; CI -1.57 to -0.618). No significant effect was found for the negative subscale. Dropout rates were not significantly different (Odds Ratio, OR 0.93; 95% CI 0.55 to 1.59). Heterogeneity was low across outcomes.

**Conclusion:**

Our findings demonstrate a statistically significant, albeit small, advantage for adjunctive CBD over placebo for total, positive, and general symptoms in schizophrenia. The lack of effect on negative symptoms suggests a targeted mechanism. Given the consistency across studies, adjunctive CBD is a promising option for specific symptom clusters, but its clinical impact requires confirmation in larger trials.

## Introduction

Cannabidiol (CBD) is a naturally occurring cannabinoid found in *Cannabis sativa* that has recently been studied as a new therapeutic option for schizophrenia and related disorders [1, 2].

CBD is a partial agonist of dopamine D2 receptors, which can contribute to its antipsychotic effects at high doses (800–1000 mg/die) [3, 4] combined with its partial agonist activity of 5HT1A receptors [5], shared with third-generation antipsychotics (aripiprazole, brexpiprazole), that can also explain the anxiolytic, antidepressant and antipsychotic effects of CBD. The pharmacodynamic profile of CBD is characterized by a low affinity for the orthosteric sites of the CB1 and CB2 receptors [6]. However, it is crucial to note that CBD exerts significant modulation at the allosteric sites of these receptors at nanomolar concentrations [7]. Studies have found that CBD can attenuate binding of CB1 agonists, thereby antagonizing CB1 signaling [7]. However, more evidence suggests the endocannabinoid system plays a more central role, particularly through the regulation of anandamide signaling. The endocannabinoid system is deeply involved in regulating dopamine release in the CNS, a mechanism linked to the release of anandamide [8]. Indeed, clinical studies have demonstrated that CBD treatment in acute schizophrenia leads to an increase in serum anandamide levels, which correlates significantly with symptom amelioration [1]. Furthermore, emerging evidence highlights a dose-dependent effect on this mechanism: a recent study indicated that doses of 800 mg/day may exert different effects on anandamide levels compared to 600 mg/day [9], suggesting that higher doses might be necessary to achieve a pronounced therapeutic effect. Finally, CBD is recognized for its neuroprotective and anti-inflammatory properties [10], which might be clinically relevant considering that neuroinflammation is an early event in the pathophysiology of schizophrenia. Unlike Δ⁹-tetrahydrocannabinol (THC), the psychoactive phytocannabinoid popular among recreational marijuana users, it does not possess psychotropic effects and is now widely studied for the treatment of organic and psychiatric disorders such as epilepsy [11] and anxiety disorders [12], due its low burden of side effects. Although the use of CBD in psychiatric disorders has already been the subject of several systematic reviews, only one meta-analysis on the effectiveness of the aforementioned compound in schizophrenia was published in 2020 [13]. While some early trials investigated CBD as a monotherapy [1], the majority of recent clinical research has focused on its use as an adjunctive treatment. This approach is primarily driven by ethical considerations, as withdrawing established antipsychotic treatment poses a significant risk of relapse and symptom exacerbation. Furthermore, adjunctive designs address the urgent clinical need to manage residual symptoms in patients with partial response to standard care. Given the key role of endocannabinoid system in schizophrenia [14] and recent evidence of other randomized controlled trials on this topic, the objective of this systematic review with pairwise meta-analysis is to update the current body of knowledge to evaluate the effectiveness of CBD as an add-on therapy in patients with schizophrenia.

## Materials and methods

### Protocol registration

The protocol for this meta-analysis was published on INPLASY, with the corresponding code INPLASY20260019. The systematic review was conducted in accordance with the Preferred Reporting Items for Systematic Reviews and Meta-Analyses (PRISMA) guidelines [15].

### Search strategy, inclusion criteria and selection process

We searched PubMed and the Cochrane Central Register of Controlled Trials (CENTRAL) up to May 14, 2025. Additionally, we reviewed the references of previously published reviews.

We applied the following inclusion criteria:

- Population: Adults aged 18–65 with a diagnosis of schizophrenia or related disorder according to the criteria of DSM IV, DSM IV-TR, DSM 5, DSM 5-TR, or ICD-10.
- Intervention: CBD as monotherapy or added to standard treatment, administered via any route and at any dose. The treatment with CBD should last at least 2 weeks.
- Comparator: Non-active placebo.
- Outcomes: Our primary outcomes were changes in total symptoms as assessed by PANSS total; changes in positive symptoms as assessed by PANSS’s positive subscale; changes in negative symptoms as assessed by PANSS’s negative subscale. Secondary outcomes were changes in anxiety and depression symptoms as well as changes in quality of life.
- Design: Randomized controlled trials, both parallel and cross-over groups.
- Language: English articles.

The selection of articles based on titles and abstracts was carried out using the Rayyan tool after the removal of duplicates by two independent authors who worked blind (ADF and PC). Conflicts were resolved through discussion between the same two authors, and in cases of irresolvable conflicts, a more experienced author (AR) was consulted. The manuscripts of the included articles were obtained and uploaded to a relational database (Airtable) to definitively assess the adherence to our inclusion criteria. Again, the evaluation was conducted in duplicate and blind by the previously mentioned two authors, using the same conflict resolution method.

### Risk of bias assessment

We conducted the risk of bias assessment blindly and in duplicate, involving authors ADF and PC, across all domains of Cochrane’s Risk of Bias 2. The evaluated domains include bias due to the randomization process, bias due to deviations from the planned intervention, bias due to missing outcome data, bias in the measurement of the outcome, and bias in the selection of the reported result. All emerging conflicts during the assessment were resolved through direct discussion between the authors or, in the case of persistent disagreement, with the involvement of another expert author (AR).

### Data extraction

The data from the articles deemed to adhere to our inclusion criteria were extracted into Airtable by two independent authors (ADF and PC), with conflicts resolved through discussion and, if necessary, the involvement of a third expert author (AR). In addition to the data within the manuscript, data were also extracted from figures using the online tool Web Plot Digitizer. In cases of missing or unclear data, the corresponding authors and/or the first authors of the original articles were contacted. Our primary outcomes were: (a) the overall efficacy of CBD as measured by the Positive and Negative Syndrome Scale (PANSS), the Brief Psychiatric Rating Scale (BPRS), or any other validated scale; (b) the positive and negative symptoms of schizophrenia as assessed by the respective subscales of the PANSS or by the Scale for the Assessment of Negative Symptoms (SANS) and the Scale for the Assessment of Positive Symptoms (SAPS) or any other validated scale.

The secondary outcomes were: (a) the levels of depressive symptoms as assessed by the Hamilton Depression Rating Scale (HAM-D), the Calgary Depression Scale for Schizophrenia (CDSS), or any other validated scale; (b) the levels of anxiety symptoms as assessed by the Hamilton Anxiety Rating Scale (HAM-A), the Beck Anxiety Inventory (BAI), or any other validated scale; (c) the drop-out rate as reported by the original studies.

### Meta-analytic approach

The statistical analysis was performed using the software R Studio with the meta package. We adopted a random-effect model. All analyses used an intention-to-treat approach whenever possible. The effect size for continuous data was the weighted mean difference (MD) when the outcome measurement scales used were the same. We calculated Hedge’s g adjusted standardized mean difference (SMD) when the psychometric scales used were different. The effect size for dichotomous outcomes was the odds-ratio (OR). Confidence intervals and p-values were calculated, with significance levels set a priori at 0.05. Heterogeneity was assessed through the inspection of forest plots along with the calculation of the I^2 statistic and its 95% confidence interval (CI). The interpretation of heterogeneity was based on the criteria proposed by Higgins and Thompson [16], where I^2 values of 25%, 50%, and 75% indicate low, moderate, and high heterogeneity, respectively. We planned a sensitivity analysis only in case of moderate or high heterogeneity but we also conducted a subgroup analysis excluding open-label trials. We planned to assess publication bias using funnel plots and to conduct Egger’s test if the criteria outlined by Sterne et al. [17] Were met, specifically if there were at least 10 studies included in the meta-analysis. No subgroup analyses or meta-regressions were performed.

## Results

### Description of included studies

**Fig. 1:**
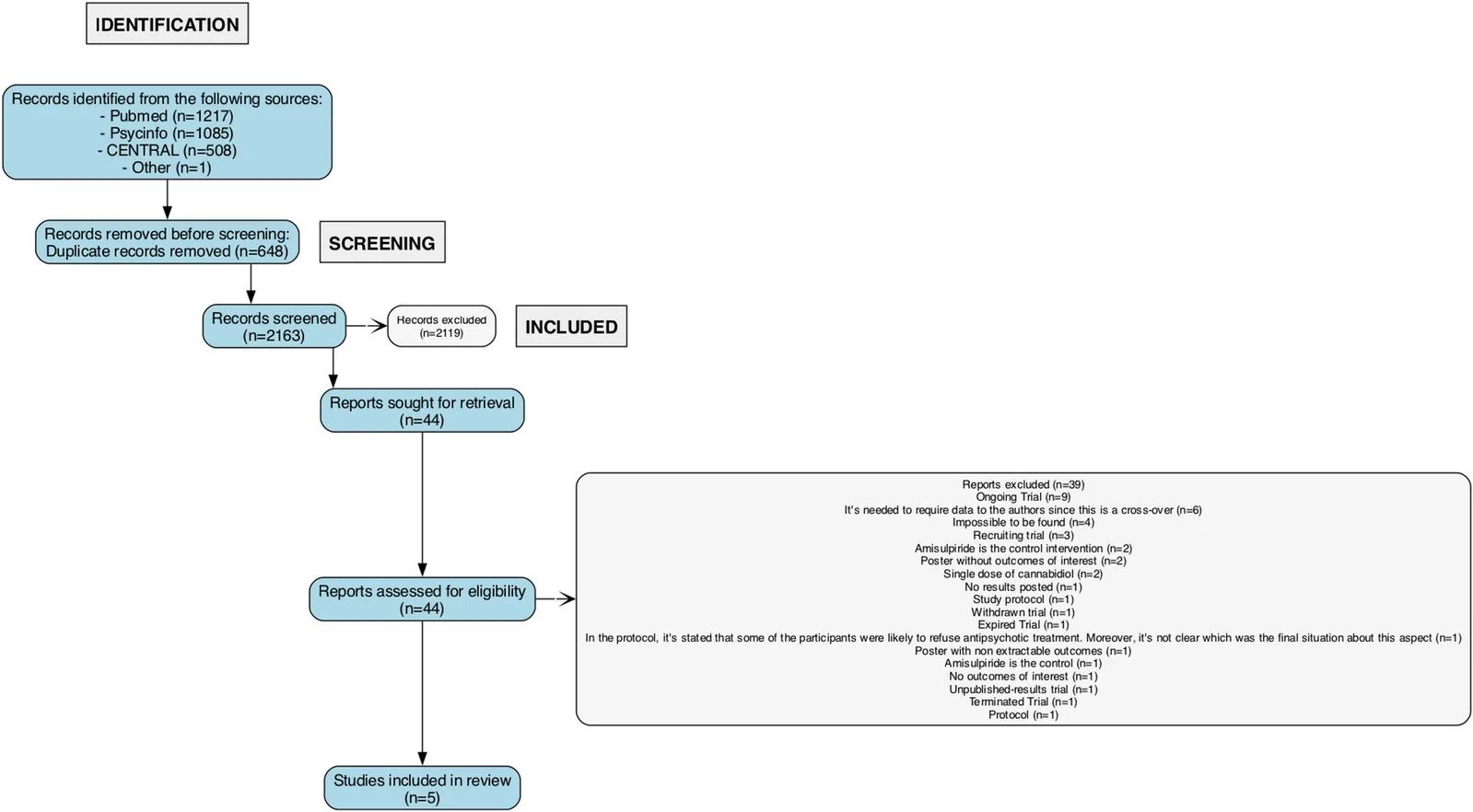
PRISMA 2020 flow diagram illustrating the study selection process. The database search identified a total of 2869 records. After removing 846 duplicates, 2163 records were screened through title and abstract reading. Of these, 44 reports were assessed in full-text for eligibility, and 39 were excluded for specified reasons. Finally, 5 studies were included in the systematic review

The search yielded 2811 results. After removing g duplicates, 648 were excluded, leaving 2163 articles for title and abstract screening. Of these, 44 articles were evaluated in full text, leading to the inclusion of 5 randomized controlled trials (see Fig. 1 for PRISMA flow-diagram). The first RCT, conducted by Boggs in 2018 (*N* = 41, mean age 47.42 ± 9.40 years, PANSS total score 79.58 ± 13.64), evaluated the efficacy of adjunctive therapy with CBD tablets at 600 mg/day compared to placebo in a randomized, controlled design for a total of 6 weeks in patients diagnosed with schizophrenia. McGuire conducted a randomized controlled trial in 2018 (*N* = 88, mean age 40.85 ± 11.76 years, PANSS total score 79.97 ± 13.76) in a pool of patients diagnosed with schizophrenia or related disorders who had partially responded to antipsychotic therapy and had been on stable treatment for at least 4 weeks. The intervention consisted of 1000 mg/day of orally administered CBD, with the control being placebo tablets, over an 8-week study duration. The last RCT included in this meta-analysis was conducted by van Boxel in 2023 (*N* = 32, mean age 26.06 ± 6.44 years, PANSS total score 53.15 ± 12.49). It included patients with schizophrenia or early-onset psychotic disorders (< 5 years) who were administered 600 mg/day of CBD in capsule form, with the comparator being placebo, over a total of 4 weeks. Finally, an open-label randomized trial conducted by Kock in 2021 (*N* = 31, mean age 35.10 ± 10.16 years, PANSS total score 79.33 ± 17.79) was included. This study included patients with both affective and non-affective psychosis and added to the use of antipsychotics by substituting tobacco cigarettes with high-CBD marijuana cigarettes (approximately 20 mg/cigarette, with an average consumption of about 9.7 CBD cigarettes per day i.e. 200 mg of CBD/day) for a total study duration of 4 weeks (see Table 1 for the full description of the characteristics of included studies). Additionally, a Phase 2 randomized, double-blind, parallel-group trial sponsored by Jazz Pharmaceuticals (formerly GW Research Ltd.), registered as NCT04421456 with results posted in 2023 (*N* = 95 enrolled participants), was considered. This study aimed to evaluate GWP42003-P (an oral CBD solution) as an adjunctive therapy compared to placebo in participants with schizophrenia (diagnosed according to DSM-5) aged between 18 and 55 years, with a baseline total PANSS score between ≥ 60 and < 110, and with an inadequate response to ongoing antipsychotic treatment. The planned intervention doses were 300 mg/day or 1000 mg/day of GWP42003-P for a treatment period of 12 weeks. However, the study was terminated prematurely based on a business decision by the sponsor.

**Table 1:** Characteristics of included studies

Study	Population	Intervention	Comparator	Duration (weeks)	Outcomes
van Boxel 2023	Adults with schizophrenia or related psychotic disorder, recent-onset (< 5 years)	CBD 600 mg/day	Placebo	4	PANSS positive subscale PANSS negative subscale PANSS general subscale PANSS total HAM-D YMRS CGI GAF SOFAS
Kock 2021	Adults with affective and non-affective psychosis	CBD cigarettes	Standard cigarettes	4	PANSS BDI SWN-K Brøset violence checklist
Boggs 2018	Adults with schizophrenia, on a standard antipsychotic dose for at least 4 weeks	CBD 600 mg/day	Placebo	6	Speed of Processing Attention/vigilance Reasoning and Problem Solving Verbal Learning Visual Learning Working Memory Social cognition PANSS total PANSS positive subscale PANSS negative subscale PANSS general subscale
McGuire 2018	Adults with schizophrenia or related psychotic disorder, at least partially responsive, stable on a dose of antipsychotic medication for the previous four weeks	CBD 1000 mg/day	Placebo	8	PANSS Total score PANSS Positive score PANSS General score PANSS Negative score SANS
NCT04421456	Adults with schizophrenia, clinically stable, PANSS Total higher than 60 and lower than 110	CBD 1000 mg/day	Placebo	12	PANSS Total score PANSS Positive score PANSS General score PANSS Negative score CGI-S CGI-I Body Weight Body Mass Index Waist Circumference Diastolic/Systolic Blood Pressure Respiratory rate Body Temperature ECGs values Number of patients with suicidal ideation or behavior

BDI: Beck Depression Inventory; CGI: Clinical Global Impression; GAF: Global Assessment of Functioning; HAM-D: Hamilton Depression Rating Scale; PANSS: Positive and Negative Syndrome Scale; SANS: Scale for the Assessment of Negative Symptoms; SOFAS: Social and Occupational Functioning Assessment Scale; SWN-K: Subjective Well-Being Under Neuroleptics Scale (short form); YMRS: Young Mania Rating Scale

* Approximately 20 mg/cigarette, with an average consumption of about 9.7 CBD cigarettes per day i.e. 200 mg of CBD/day

### Risk of bias assessment

**Fig. 2:**
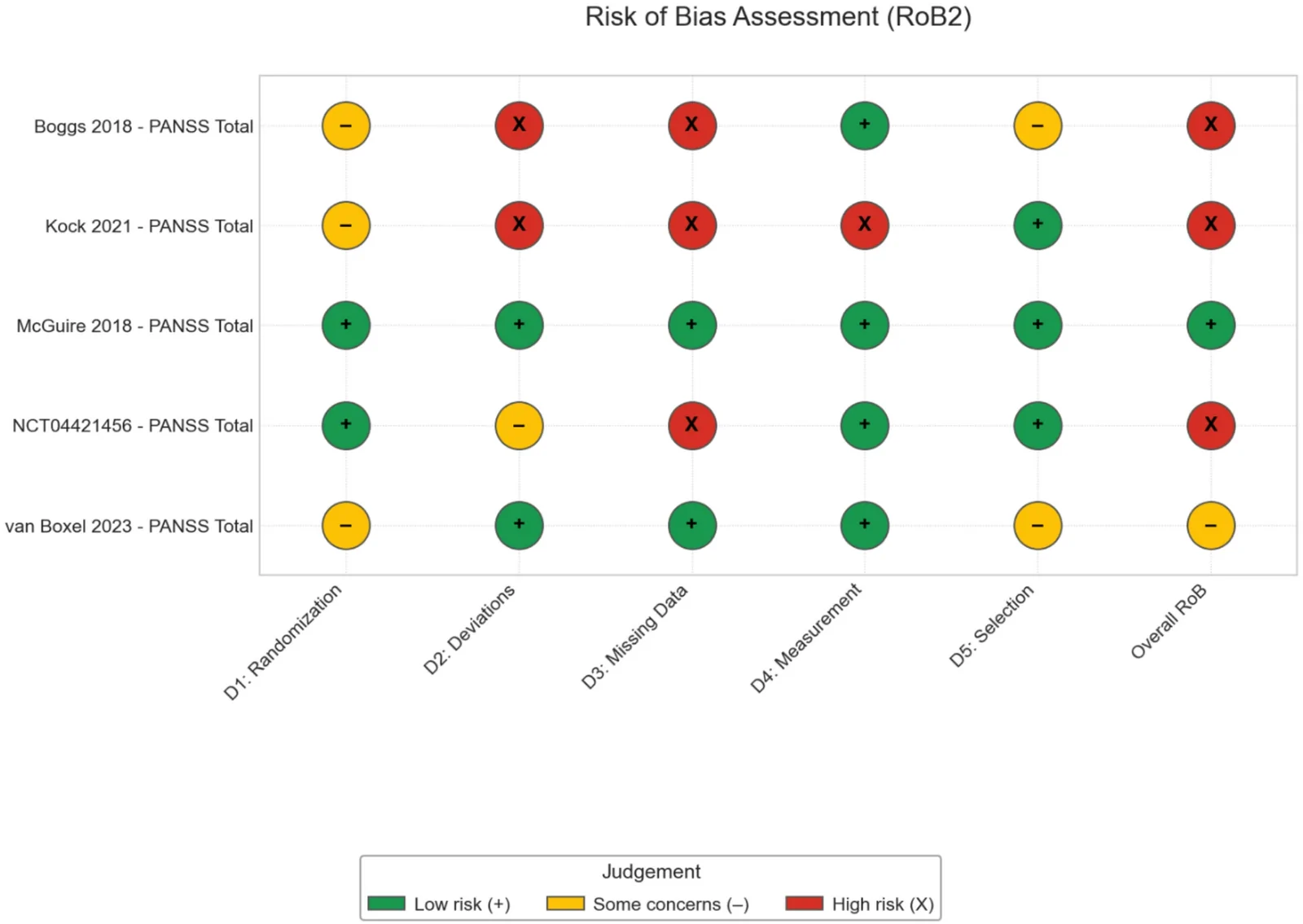
Risk of bias assessment (RoB2) for the included studies. The chart summarizes the risk of bias for each study according to the five domains of the RoB2 tool: (D1) randomization process, (D2) deviations from intended interventions, (D3) missing outcome data, (D4) measurement of the outcome, and (D5) selection of the reported result. The overall judgment (Overall RoB) for each study is shown in the final column

In our study, we conducted a risk of bias assessment for the articles included in the meta-analysis using the Cochrane Risk of Bias 2 tool. This tool examines various domains, including random sequence generation, allocation concealment, blinding of participants and personnel, blinding of outcome assessment, incomplete outcome data, selective reporting, and other potential sources of bias. The analysis revealed a varied landscape in terms of the methodological quality of the included studies. McGuire’s 2018 [18] study stood out for its low risk of bias in almost all evaluated domains. This double-blind RCT demonstrated solid methodology, with randomization managed by an external statistician, appropriate allocation concealment, and the use of both intention-to-treat and per-protocol analyses. Additionally, data were available for over 95% of the patients, and the study protocol was consistent with the reported data. The only minor concern was the lack of a detailed explanation of the randomization process, but this was not considered sufficient to increase the overall risk level. On the other hand, Boggs’ 2018 [19] study presented several issues leading to a high risk of bias assessment. Despite being described as a double-blind RCT, information on the randomization process and allocation concealment was insufficient. The study likely used a per-protocol approach, excluding dropouts from the analysis, which could introduce bias. About 10% of patients were excluded, and data for 4 participants were not reported without adequate explanation. Kock’s 2021 [20] study also showed a high risk of bias. As an open-label study, there were significant concerns regarding blinding, exacerbated by the intense smell of CBD cigarettes, making true blinding of the intervention impossible. The study used a per-protocol approach and had a high dropout rate, with data reported for only 7 patients per arm. Some participants continued using THC cannabis during the study, and the CBD dose was low (compared to other studies) and variable, introducing additional sources of bias. Finally, van Boxel’s 2023 [21] study was assessed with some concerns in its risk of bias assessment. Although it was a double-blind RCT with low attrition (only one patient withdrew consent), there was insufficient information on the randomization process and allocation concealment. The main concern was that the study protocol, although mentioned by the authors, was not available for verification. The NCT04421456 study showed a mixed risk profile. It demonstrated low risk of bias in three domains: randomization process (D1), measurement of outcome (D4), and selection of reported results (D5). However, it had some concerns risk of bias for deviations from intended interventions (D2) and high risk regarding missing data (D3). In conclusion, this risk of bias assessment reveals considerable variability in the methodological quality of the studies included in our meta-analysis. While McGuire’s study provides more reliable data due to its low risk of bias, the studies by Boggs and Kock present significant limitations that could affect the interpretation of their results. Van Boxel’s study falls in an intermediate position, with some concerns mainly due to the lack of access to the study protocol. For an overview of the risk of bias characteristics in the studies, please refer to Fig. 2.

### Comparison – CBD augmentation vs. placebo

**Fig. 3:**
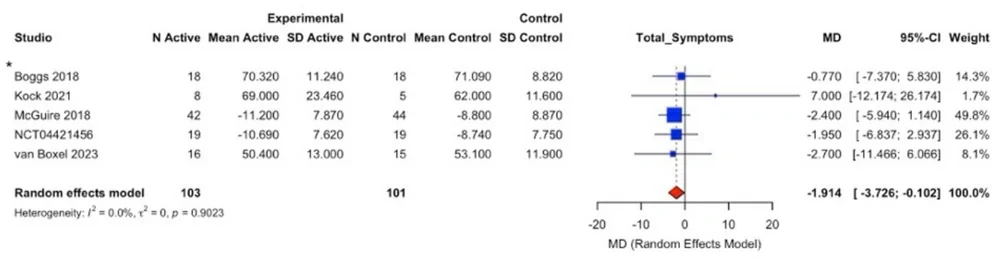
Forest plot of the meta-analysis for total symptoms. The pooled estimate of the studies shows a mean difference (MD) of -1.914 (95% CI [-3.726, -0.102]), indicating a statistically significant overall effect in favor of the experimental group. Heterogeneity was absent (I2 = 0%). *For the Boggs 2018 study, the dispersion values were identified as standard errors (SE) instead of standard deviations (SD), as reported by the authors. This correction was applied because the original values were clearly too small to represent the SD and consistent with the expected magnitude for an SE, thus ensuring appropriate weighting of the study in the meta-analysis

**Fig. 4:**
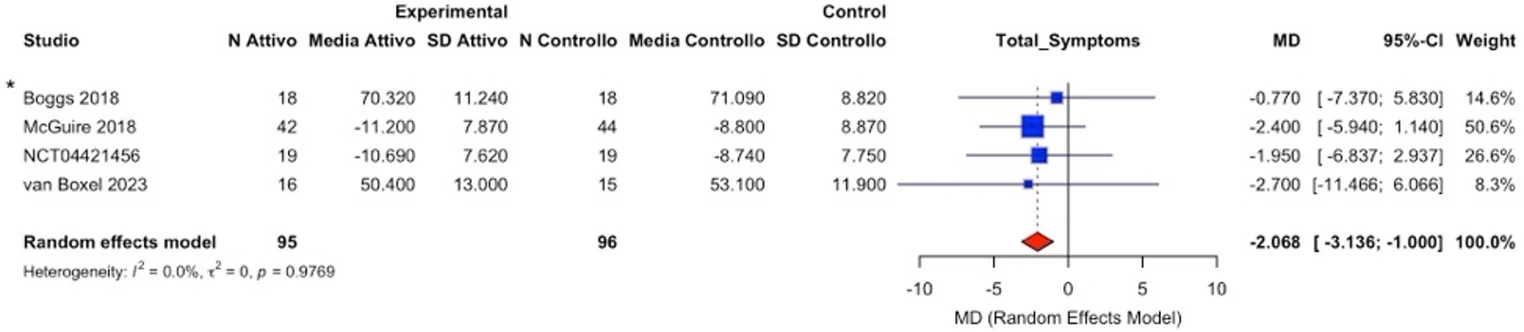
Forest plot of the sensitivity analysis for total symptoms, excluding the Kock 2021 study. The reanalysis of the data shows an aggregated mean difference (MD) of -2.068 (95% CI [-3.136, -1.000]), confirming a statistically significant effect in favor of the experimental group even after the exclusion of the study. The heterogeneity remained zero (I2 = 0%). *For the Boggs 2018 study, the dispersion values were identified as standard errors (SE) instead of standard deviations (SD), as reported by the authors. This correction was applied because the original values were clearly too small to represent the SD and consistent with the expected magnitude for an SE, thus ensuring appropriate weighting of the study in the meta-analysis

**Fig. 5:**
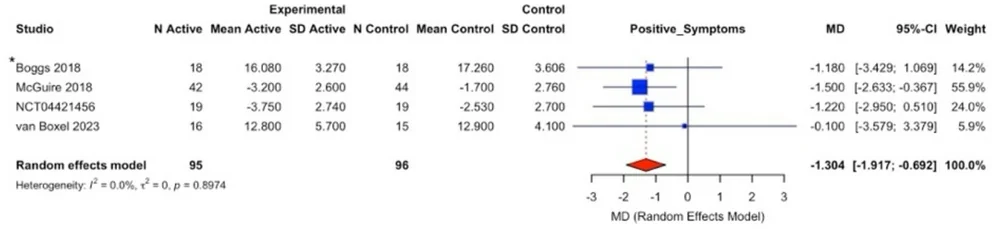
Forest plot of the meta-analysis for PANSS Positive. The diamond represents the aggregated mean difference (MD), which is -1.304 (95% CI [-1.917, -0.692]), indicating a statistically significant effect in favor of the experimental group. The heterogeneity among the studies was found to be zero (I2 = 0%). *For the Boggs 2018 study, the dispersion values were identified as standard errors (SE) instead of standard deviations (SD), as reported by the authors. This correction was applied because the original values were clearly too small to represent the SD and consistent with the expected magnitude for an SE, thus ensuring appropriate weighting of the study in the meta-analysis

**Fig. 6:**
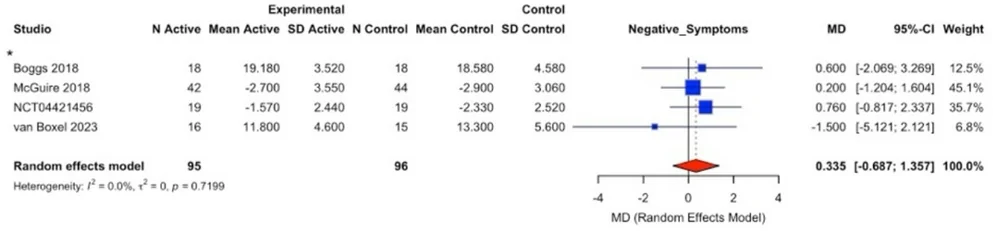
Forest plot of the meta-analysis for PANSS Negative. The diamond, indicating the aggregated estimate, shows a mean difference (MD) of 0.335 (95% CI [-0.687, 1.357]). Since the confidence interval crosses zero, the resulti s not statistically significant. The heterogeneity among the studies is zero (I2 = 0%). *For the Boggs 2018 study, the dispersion values were identified as standard errors (SE) instead of standard deviations (SD), as reported by the authors. This correction was applied because the original values were clearly too small to represent the SD and consistent with the expected magnitude for an SE, thus ensuring appropriate weighting of the study in the meta-analysis

**Fig. 7:**
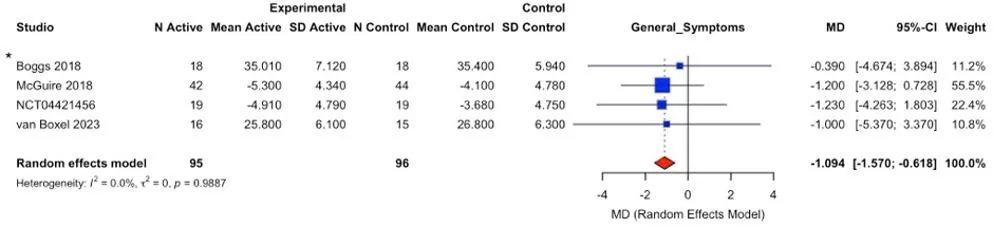
Forest plot of the meta-analysis for PANSS General. The pooled mean difference (MD), represented by the diamond, is -1.094 (95% CI [-1.570, -0.618]), showing a statistically significant result that favors the experimental treatment. No heterogeneity was found among the studies (I2 = 0%). *For the Boggs 2018 study, the dispersion values were identified as standard errors (SE) instead of standard deviations (SD), as reported by the authors. This correction was applied because the original values were clearly too small to represent the SD and consistent with the expected magnitude for an SE, thus ensuring appropriate weighting of the study in the meta-analysis

This meta-analysis evaluated the efficacy of the CBD augmentation compared to a placebo on symptomatology assessed by the Positive and Negative Syndrome Scale (PANSS), utilizing a random-effects model for all analyses. For the PANSS Total score, an initial analysis including all five studies, encompassing 204 participants (103 experimental, 101 control), indicated a statistically significant reduction in the intervention group compared to controls (Mean Difference, MD = -1.914; 95% Confidence Interval [CI] = [-3.726, -0.102], Fig. 3), with no heterogeneity observed (I^2^ = 0.0%, *p* = 0.9023). A post-hock subgroup analysis for the PANSS Total score, including only the four double-blind randomized controlled trials (RCTs: Boggs 2018, NCT04421456, McGuire 2018, van Boxel 2023) with 191 participants (95 experimental, 96 control), also demonstrated a statistically significant reduction in the intervention group (MD = -2.068; 95% CI = [-3.136, -1.000], Fig. 4), again with no heterogeneity (I^2^ = 0.0%, *p* = 0.9769). Regarding the PANSS Positive subscale, data from the four RCTs (191 participants) showed a significant reduction in positive symptoms for the intervention group (MD = -1.304; 95% CI = [-1.917, -0.692], Fig. 5), with no heterogeneity (I^2^ = 0.0%, *p* = 0.8974). Conversely, the analysis of the PANSS Negative subscale, based on these four RCTs (191 participants), did not reveal a statistically significant difference between the intervention and control groups (MD = 0.335; 95% CI = [-0.687, 1.357], Fig. 6), with the confidence interval crossing zero and no heterogeneity detected (I^2^ = 0.0%, *p* = 0.7199). Finally, for the PANSS General Psychopathology subscale, the pooled results from the four RCTs (191 participants) indicated a statistically significant improvement in the intervention group (MD = -1.094; 95% CI = [-1.570, -0.618], Fig. 7), with no heterogeneity present (I^2^ = 0.0%, *p* = 0.9887). Across all reported analyses, statistical heterogeneity as measured by I^2^ was consistently 0.0%, and the p-values from Cochran’s Q test were non-significant (*p* > 0.05), indicating homogeneity in effect estimates across the included studies for each outcome.

### Drop outs

**Fig. 8:**
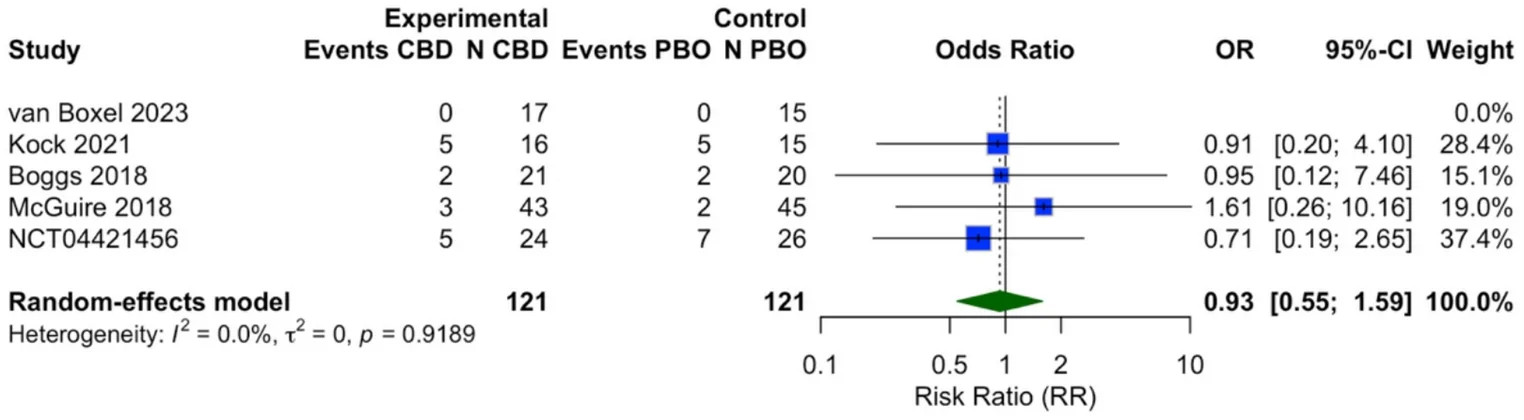
Forest plot for the meta-analysis of total dropouts. The pooled analysis shows no statistically significant difference in the odds of all-cause dropout between the experimental (CBD) and control (PBO) groups. The pooled Odds Ratio (OR) was 0.93 (95% CI [0.55, 1.59]). No heterogeneity was detected among the studies (I2 = 0%)

**Fig. 9:**
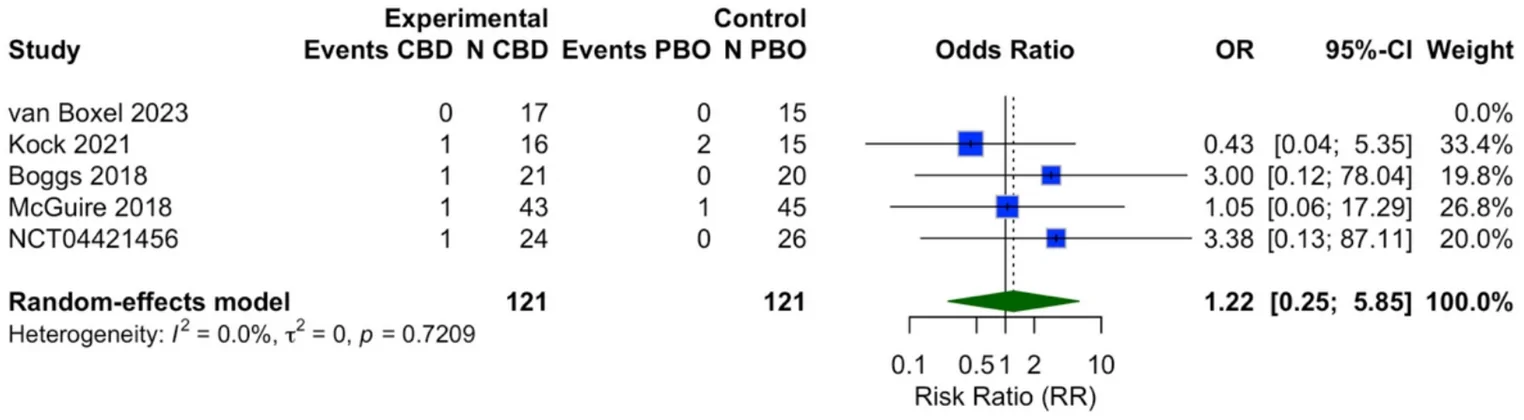
Forest plot for the meta-analysis of dropouts due to adverse events. The diamond represents the pooled Odds Ratio (OR), which was 1.22 (95% CI [0.25, 5.85]). The result is not statistically significant, as the confidence interval crosses 1, indicating no significant difference in the odds of dropping out due to adverse events between the two groups. There was no evidence of heterogeneity (I2 = 0%)

In addition to evaluating symptomatic efficacy, the meta-analysis examined study dropout rates as an indirect measure of the overall tolerability of adjunctive CBD treatment compared to placebo. Two separate analyses were conducted: one for dropouts due to any cause and one specific to dropouts due to adverse events. In the analysis of all-cause dropouts, pooled data from 5 studies were included, totaling 121 participants in the experimental (CBD) group and 121 in the control (placebo) group. The random-effects model showed an overall Odds Ratio (OR) of 0.93, with a 95% Confidence Interval (CI) ranging from 0.42 to 2.08 (Fig. 8). Since the confidence interval includes the value 1.0, this difference was not statistically significant, suggesting that the addition of CBD did not significantly alter the overall probability of dropping out of the study compared to placebo. Heterogeneity among studies for this outcome was nil (I^2^ = 0.0%, *p* = 0.9189). Similarly, a specific analysis was conducted for dropouts caused by adverse events, including data from the same 5 studies with 121 participants per group. The pooled OR was 1.22 (95% CI: 0.28 − 5.19, Fig. 9). In this case as well, the confidence interval crosses the value 1.0, indicating the absence of a statistically significant difference between the CBD group and the placebo group regarding study dropout specifically due to side effects. Heterogeneity was again nil (I^2^ = 0.0%, *p* = 0.7204). Overall, these analyses on dropout rates suggest that adding CBD to standard antipsychotic treatment was generally well-tolerated, showing no significant increase in the risk of study discontinuation compared to placebo, neither for general causes nor specifically for adverse events. This finding is consistent with the good tolerability profile of CBD reported in the literature.

## Discussion

Our study examined the efficacy of cannabidiol (CBD) as an adjunctive therapy in patients with schizophrenia by analyzing the results of five randomized controlled trials. Our updated meta-analysis found statistically significant reductions favoring the CBD-treated group compared to placebo for the PANSS total score, the PANSS positive subscale, and the PANSS general psychopathology subscale. No significant differences were found for the PANSS negative subscale or for the dropout rate. However, it is crucial to interpret these statistically significant results with caution. They are based on a still limited number of studies (four double-blind RCTs plus one open-label study) and a relatively low total number of participants, limiting the generalizability of the results, although statistical heterogeneity among studies for these outcomes was low. Furthermore, the duration of the included studies (4–8 weeks) may not be sufficient to fully capture the potential benefits of CBD, especially those related to its long-term neuroprotective or anti-inflammatory effects. Despite these limitations, CBD has generated considerable interest due to its unique multimodal pharmacodynamic profile [4], demonstrating anxiolytic and antipsychotic properties in animal models [22, 23]. Preclinical studies suggest interactions with the endocannabinoid, serotonergic [5], dopaminergic [3], and glutamatergic [24] systems, all implicated in the pathophysiology of schizophrenia [25, 26]. Moreover, its anti-inflammatory and neuroprotective properties [10, 27] could be relevant, given the growing evidence of the role of neuroinflammation in schizophrenia. Nevertheless, challenges remain in interpreting the gap between preclinical potential and clinical benefits, even those now statistically significant observed in our analysis. The heterogeneous complexity of schizophrenia might play a role, making it possible that CBD is more effective in specific subgroups (e.g., patients in their first psychotic episode or at high risk [28–30]. The doses used (ranging from ~ 200 to 1000 mg/day) might also not yet be optimal [31]. Crucially, pharmacokinetic factors were not considered in the included studies. The oral bioavailability of CBD is known to be low and highly variable, strongly influenced by food; this uncontrolled variability could have altered the results. Furthermore, CBD is metabolized by and inhibits hepatic cytochrome P450 enzymes, creating significant potential for unmonitored drug interactions with concomitant antipsychotics, which could alter plasma levels of both drugs and thus efficacy or tolerability. These pharmacokinetic and interaction considerations were not adequately addressed in the included studies. Despite these important methodological and pharmacokinetic limitations in current studies, the trend towards benefit, now supported by statistical significance for total, positive, and general symptoms, and the good tolerability profile of CBD [32], suggest that research on CBD as an adjunctive therapy remains a promising area.

## Conclusions

In conclusion, this updated meta-analysis provides new statistically significant evidence, albeit based on limited data, for the efficacy of adjunctive CBD in reducing certain symptomatic domains of schizophrenia. The good tolerability and positive trends suggest that CBD remains an interesting candidate.

Larger, multicenter studies with longer durations and optimized dosages are needed in the future. It is crucial that these studies incorporate pharmacokinetic evaluations (monitoring plasma levels of CBD and concomitant drugs) and consider the impact of diet on bioavailability, to address current significant limitations and better understand the true therapeutic potential of CBD. Investigating the efficacy of CBD in specific subgroups (e.g., first psychotic episode, high risk, inflammatory profile) could further clarify its role. Research on CBD in schizophrenia deserves to be pursued with more methodologically robust studies.

## Data Availability

Data used for this study are available under reasonable request to Dr. Antonio Di Francesco.
